# A Milled Microdevice to Advance Glia-Mediated Therapies in the Adult Nervous System

**DOI:** 10.3390/mi10080513

**Published:** 2019-07-31

**Authors:** Juan S. Peña, Denise Robles, Stephanie Zhang, Maribel Vazquez

**Affiliations:** 1 Department of Biomedical Engineering, Rutgers, The State University of New Jersey, New Brunswick, NJ 08901, USA; 2 Department of Biomedical Engineering, State University of New York at Binghamton, Binghamton, NY 13902, USA

**Keywords:** Müller glial cells, Schwann cells, microfluidics, chemotaxis, computer numerical controlled (CNC), neurotrophic factors

## Abstract

Neurodegenerative disorders affect millions of adults worldwide. Neuroglia have become recent therapeutic targets due to their reparative abilities in the recycling of exogenous neurotoxins and production of endogenous growth factors for proper functioning of the adult nervous system (NS). Since neuroglia respond effectively to stimuli within in vivo environments on the micron scale, adult glial physiology has remarkable synergy with microscale systems. While clinical studies have begun to explore the reparative action of Müller glia (MG) of the visual system and Schwann Cells (ShC) of the peripheral NS after neural injury, few platforms enable the study of intrinsic neuroglia responses to changes in the local microenvironment. This project developed a low-cost, benchtop-friendly microfluidic system called the glia line system, or gLL, to advance the cellular study needed for emerging glial-based therapies. The gLL was fabricated using elastomeric kits coupled with a metal mold milled via conventional computer numerical controlled (CNC) machines. Experiments used the gLL to measure the viability, adhesion, proliferation, and migration of MG and ShC within scales similar to their respective in vivo microenvironments. Results illustrate differences in neuroglia adhesion patterns and chemotactic behavior significant to advances in regenerative medicine using implants and biomaterials, as well as cell transplantation techniques. Data showed highest survival and proliferation of MG and ShC upon laminin and illustrated a four-fold and two-fold increase of MG migration to dosage-dependent signaling from vascular endothelial growth factor (VEGF) and epidermal growth factor (EGF), respectively, as well as a 20-fold increase of ShC migration toward exogenous brain-derived neurotrophic factor (BDNF), compared to media control. The ability to quantify these biological parameters within the gLL offers an effective and reliable alternative to photolithography study neuroglia in a local environment ranging from the tens to hundreds of microns, using a low-cost and easily fabricated system.

## 1. Introduction

**Figure 1:**
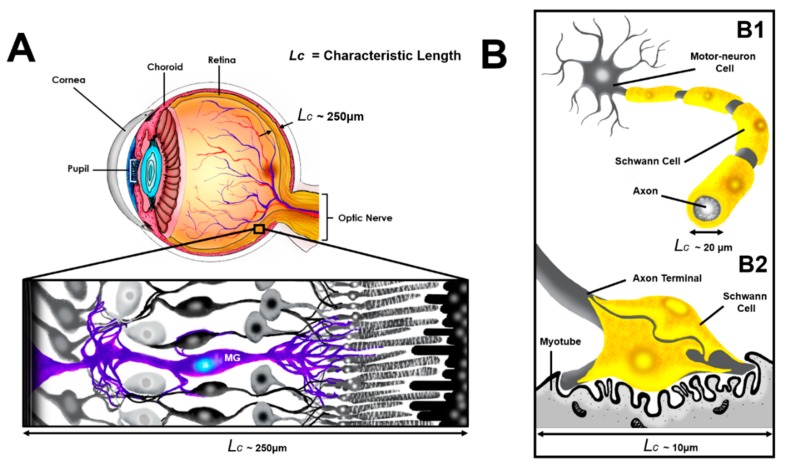
Schematic of glial cells from the central and peripheral nervous system. (**A**) Rendering of the human eye from the posterior (right) to anterior (left) with an inset of retinal tissue. Inset displays the cellular interaction between photoreceptors, horizontal, bipolar, amacrine and ganglion neurons, and Müller glia cells (purple) across an approximate 250 µm thickness. (**B**) Motor neuron (MN) interaction with Schwann cells (ShC) at the neuromuscular junction. (**B1**) ShC myelination along MN axon. (**B2**) Peri-synaptic ShC at the MN axon terminal of the neuromuscular junction [9]. *L_c_* refers to the characteristic length of the local microenvironment where glial cells reside.

Degenerative neural disorders lead to progressive failure of motor function, sensation, and/or vision in millions of adults worldwide [1]. Neuroglia have become recent therapeutic targets because of their regenerative capabilities in repairing damage across the adult nervous system (NS) [2,3]. The scale of adult glial physiology has remarkable synergy with larger microscale systems, as neuroglia respond effectively to stimuli within in vivo microenvironments on the order of tens to hundreds of microns, rather than single or sub-micron stimuli often employed for single-cell analyses [4]. As per Figure 1A, Müller glia (MG) of the visual system provide structural and trophic support for the diverse cohort of retinal neurons required for vision [5,6,7,8], while Schwann glial cells (ShC) of the peripheral nervous system (PNS) modulate biochemical synapses for somatic movement [9,10] and axonal myelination [11,12]. Müller glia traverse all retinal layers with characteristic length (*L_c_*) of approximately 250 μm, monitoring the homeostasis and contributing to the structure of the retina in different retinopathies. When injury is present, Müller glia migrate toward the affected area, rapidly proliferate, and create a glia scar on the range of *L_c_* scale [13,14]. Similarly, each peripheral nerve is composed of several fascicles that contain bundles of motor neuron axons, which serve to receive and transduce sensory inputs as per Figure 1B. The diameter of peripheral nerve axons is in the scale of tens of microns, while the characteristic thickness of fascicles lies in the hundreds of microns. Transplantation of MG and ShC has prolonged the survival and axonal branching of retinal photoreceptors in dystrophic rats [15,16,17,18,19,20,21,22], as well as PNS neurons, post-spinal cord injury [23,24,25,26,27,28]. Experimental testing platforms able to evaluate and predict the reparative behavior of glia in the adult NS will dramatically advance regenerative cell therapies. 

Microsystems used for the quantitative scrutiny of neuroglia by creating chemical, electrical, and physical stimulation are underexplored. Microfluidic systems used for neuroglia applications remain surprisingly scarce despite their ability to facilitate characterization of glia responses to customized biomaterials [29], pharmacological compounds [30], and electro-chemical fields currently being explored to aid neurorepair [31]. While a slower adaptation of microsystems may be attributed to the high costs associated with clean room facilities [32] and/or the perceived need for engineering expertise to design and troubleshoot complex systems [33], the rising availability of fabrication techniques such as 3D printing/rapid prototyping [34], paper microfluidics [35,36], and toner or inkjet printing [37,38] have greatly reduced the barriers to entry in the usage of larger microscale tools (reviewed in [39]). These synergies now enable biomedical researchers to generate microsystems customized for glia-based research. 

The current report describes the application of a benchtop-friendly and cost-effective microfluidic system called the glia-line, or gLL, to bolster translational glia research. The microdevice operates using familiar benchtop protocols, independent of recycling fluidic baths or specialized electrical circuitry and can be incubated for several hours prior to, during, and after cell visualization and testing. Further, the system is generated using commercial elastomeric molding kits in tandem with a low-cost, milled mold rapidly produced by conventional computer numerical control (CNC) instruments. The gLL scale enables diverse applications, such as the inclusion of neighboring cell types to characterize cell-to-cell interactions, originally to the local in vivo microenvironment, as shown in Figure 1A,B. Müller glial cells can be readily cultured with retinal neurons or Schwann cells can be coupled with motor neurons within the gLL to aid the study of cellular behavior and promote therapeutic efforts for degenerative models. In this work, gLL systems were used to evaluate the viability and adhesion of glia upon different extracellular matrix (ECM) compounds of poly-L-lysine (PLL), laminin (LM), and collagen I (CL), as well as glia migration in response to dosage-dependent signaling from epidermal growth factor (EGF), brain-derived neurotrophic factor (BDNF), and vascular endothelial growth factor (VEGF), which are key factors in neuropathies of the visual and peripheral NS. Results illustrate statistical differences in cell survival and morphology in gLL systems over time compared to controls, as well as changes in intrinsic cell motilities, demonstrating the utility of this system in advancing glia-aided treatments for degenerative neural disorders.

## 2. Materials and Methods

### 2.1. Cell Culture

Müller Glia Cells (MG) were represented by cultured rMC-1 cells (Kerafast, Cat. No. ENW001), an immortalized MG cell line derived from a rat model and used extensively in retinal study [40,41,42,43]. Cells were maintained in Dulbecco’s Modified Eagle’s medium (DMEM) (Cat. No. 30-2002, ATCC, Manassas, VA, USA) containing 4 mM L-glutamine, 4500 mg/L glucose, 1 mM sodium pyruvate, and 1500 mg/L sodium bicarbonate supplemented with 10% fetal bovine serum (FBS) (Invitrogen-Gibco, Rockville, MD, USA) at 37 °C and 5% CO_2_. Two weeks prior to seeding within the gLL, cells were cultured in serum-restricted media (1% FBS in DMEM) at a concentration of 1.0 × 10^6^ cells/mL in T-75 flasks, as per conventional protocols [43]. Schwann glial cells (ShC) were represented by cultured S42 cells (ATCC CRL-2942), an immortalized cell line derived from the rat sciatic nerve used extensively in PNS study [44,45]. ShC were thawed, plated, and cultured in sterile Dulbecco’s modified eagle medium (DMEM, ATCC Cat. No. 30-2002) containing 10% FBS and 1% penicillin-streptomycin [45]. Cells were cultured (37 °C, 95% humidity, 5% CO_2_) and passaged (80%–90% confluency) on sterile tissue culture treated petri dishes at a density of 500–1000 cells per cm^2^.

### 2.2. Reagents

Experiments of this study examined cellular adhesion and migration on various substrates. gLL systems were functionalized with different extracellular matrices (ECM): (i) PLL: 15 µg/mL of Poly-L-Lysine (Cat. No. 25988-63-0, Sigma-Aldrich, St. Louis, MO, USA) diluted in DPBS; (ii) LM: 15 µg/mL of Laminin (Cat. No. 23017015, Life Technologies Corporation, IL) diluted in DPBS; and (iii) CL1: 10 µg/mL of Collagen type I (Cat. No. 9007-4-5, Sigma Aldrich, GA) diluted in 0.1% acetic acid (BD Biosciences, Bedford, MA, USA). In addition, cell migration was measured in response to external environments of different neurotrophic factors: (i) EGF: 100 ng/mL of epidermal growth factor (Cat. No. SRP3196, Sigma Aldrich); (ii) VEGF: 100 ng/mL of vascular endothelial growth factor (Cat. No. SRP3182, Sigma Aldrich); (iii) and BDNF: 100 ng/mL of brain-derived neurotrophic factor (Cat. No. 248-BD-005, R&D Systems, Minneapolis, MN, USA).

### 2.3. The Glia-Line System (gLL)

#### 2.3.1. Design of the gLL 

**Figure 2:**
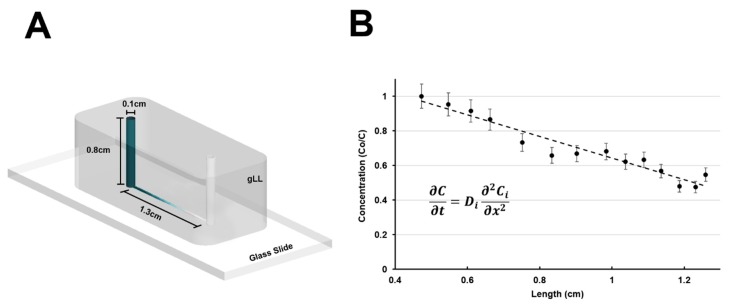
Structural design of the glia line system (gLL) alongside transport analysis in the microchannel. (**A**) CAD illustration of the gLL system, featuring a 1.3 cm long microchannel and two 6.3 μL reservoirs of 0.8 cm height and 0.1 cm diameter. (**B**) Experimental measurements of diffusion of Dextran (MW = 10 kDa) across the microchannel. Plot points represent the intensity values collected after a period of 24 h and a best-fit line model to the analytical solution of Fick’s Law. (R^2^ = 0.9317).

The gLL consists of two cylindrical reservoirs of 1 mm in diameter, 0.8 cm in height, and 6.3 µL volume each, both connected by a microchannel that is 1.3 cm in length (*ℓc*), 180.75 ± 4.71 µm in height, and 207.25 ± 6.57 µm in width, which is the equivalent of 193.1 µm in hydraulic diameter (*D_h_*) [46], as per Figure 2A. Reagents are inserted into one reservoir and transport occurs along the microchannel toward the opposite reservoir via one-dimensional diffusion, defined by Fick’s Law:

(1) $$\frac{\partial C}{\partial t}=D\frac{\partial^{2}C}{\partial x^{2}}$$

where *C* (kg/m^3^) is the solution concentration, *t* (seconds) is time, *D* (m^2^/s) denotes the diffusion coefficient of the reagent of interest, and *x* represents the direction of diffusional flow [47].

#### 2.3.2. Transport Modeling and Validation

As a highly tunable microfluidic device, the gLL enables for both static and continuous flow rate. Static condition can be accomplished by establishing an equilibrated hydrostatic pressure in the device, easily done by connecting the fluid between the two reservoirs. Continuous flow rate can be modulated via a syringe pump, with adequate tubing connecting the syringe from the pump and the source reservoir of the gLL. Static flow enables seeding of cells on substrates to examine cellular adhesion, morphology and migration over time. Continuous flow generated via a syringe pump can be tailored to mimic interstitial fluid flow to examine the effects of shear-induced flow. In this work, one-dimensional transport of targeted growth factors (EGF, VEGF, and BDNF) within the gLL was computationally modeled (COMSOL Multiphysics 5.3a, COMSOL Inc., Stockholm, Sweden) to predict reagent concentration over time along the microchannel. In these simulations, the diffusion coefficient, D, was estimated to be 2.0 × 10^−6^ cm^2^/s for EGF [48], 9.0 × 10^−7^ cm^2^/s for VEGF, and 1.4 × 10^−6^ cm^2^/s for BDNF [49]. The diffusion coefficients for VEGF and BDNF were determined by calculating the Stokes radius according to reagent molecular weight [49] and using the Stokes–Einstein equation. Development of a linear quasi-steady state concentration gradient within the gLL microchannel was experimentally validated via time-lapse imaging of fluorescein isothiocyanate-dextran (Cat. No. 0060842-46-8, 10kDa, Sigma-Aldrich) diffusion [49]. A quasi-steady-state is used when a dependent variable, in this case time, can be regarded as constant or at steady state with respect to the instantaneous values of concentration [50]. Dextran was diluted in PBS 1X to a working concentration of 50 mg/mL, after which 50 µL of this solution was inserted into one reservoir, and allowed to diffuse across the microchannel for 24 h. Images of the diffused dextran along the 1.3-cm-long microchannel were captured in intervals of 100 µm intervals, as previously performed by our group [51,52]. Average intensity values were analyzed using ImageJ (NIH Shareware, 8-bit: 1-255, Bethesda, MA, USA) and normalized to the inlet concentration, *C*_0_, as seen in Figure 2B. All cell experiments were performed post steady-state. 

#### 2.3.3. Manufacture and Assembly

**Figure 3:**
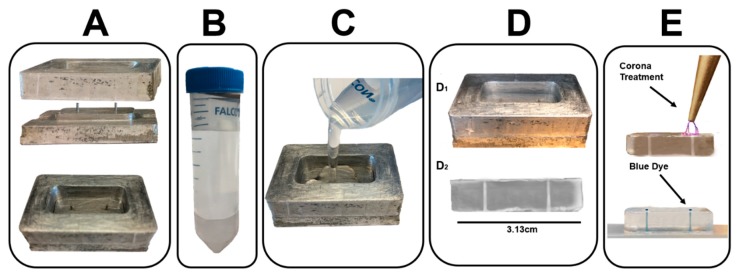
Key steps in the fabrication of the gLL system. (**A**) A desired pattern is machined into an aluminum mold via conventional computer-numerical controlled (CNC) machining. (**B**) A polydimethylsiloxane (PDMS) mixture is prepared using a 9:1 ratio of elastomer base to curing agent (weight per volume). (**C**) The PDMS mixture is poured into the machined mold of the gLL to reach the height of machined rods. (**D**) gLL casting using soft lithography techniques. (**D1**) PDMS is cured in a convection oven. (**D2**) Image of gLL cast from mold. (**E**) Corona treatment applied to gLL bonded to a glass slide. The gLL device was filled with blue dye to highlight its fluidic chambers, reservoirs, and microchannel.

The gLL is cast in commercial polydimethylsiloxane (PDMS; Cat. No. 1020992-312, VWR, PA, USA) and bonded to a chemically cleaned (piranha etch) microscope slide. As shown in Figure 3, the gLL was developed using two separate techniques: (i) Fabrication of a milled, aluminum mold via computer numerical control (CNC) and (ii) elastomeric molding using commercial polydimethylsiloxane (PDMS; Cat. No. 1020992-312, VWR, PA) with an elastomer base to curing agent ratio of 1:9. The 3-axis TRAK DPM SX2P Bed Mill with the ProtoTRAK SMX CNC was used to mill the gLL design on aluminum, with a tolerance range of ±40 µm. The configurations and specifications of the CNC milling processes are summarized in Table 1. The CNC-fabricated mold is comprised of two layers that mechanically assemble atop one another. The bottom layer contains a base with two rods of 0.1 mm in diameter and 0.8 cm in height each, connected by a machined microchannel of 1.3 cm in length, 214.0 µm in width, and 217.5 µm in height. The top layer consists of a hollow rectangular structure that is press fitted onto the bottom layer to enclose the device. 

The elastomer was formed using a solution of PDMS in 9:1 ratio (weight per volume) with its curing agent that was mixed and vacuum desiccated for 15 min to remove excess bubbles. After degassing, approximately 5 mL of the mixture was poured into the milled aluminum mold and allowed to polymerize in a convection oven (T = 105 °C) for 15 min. Once polymerized, the elastomer was manually removed from the machined mold and exposed to corona plasma treatment for 30 s. The elastomer was then firmly pressed upon a microscope glass slide, which had previously been chemically cleaned via piranha etching and also corona-treated for 30 s to generate an ozone-bonded, closed gLL system. The surface-relief of the aluminum mold and PDMS elastomer was measured using a Mitutoyo PH-3500 profile projector coupled with the QM-Data 200 optical measurement data processing system. This equipment quantified the geometry of gLL’s CNC-machined microchannel mold and the dimensions of the cast PDMS gLL device.

#### 2.3.4. gLL Operation

Multiple gLL devices were individually treated with different ECM substrates of poly-L-lysine (PLL), laminin (LM), and collagen type I (CL1). Approximately 100 µL of each substrate was manually loaded into the devices via syringe and allowed to crosslink at 37 °C overnight within a 5% CO_2_ incubator. Once substrate crosslinking occurred, excess ECM solution within the microchannel was aspirated and the microchannel cleaned by flushing PBS via syringe from one reservoir to another to validate fluid flow. Cultured cells were dislodged using Accutase^®^ Solution (Cat. No. 10210-214, VWR, PA), re-suspended in 10% FBS supplemented DMEM at a density of 1.0 × 10^6^ cells/mL, and manually inserted into gLL devices using a syringe. 

### 2.4. Measurement of Cell Viability, Proliferation, and Morphology 

MG and ShC were seeded and incubated at 37 °C for 24 h for attachment within ECM-coated gLL devices. gLL systems without substrate coatings on microchannel surfaces were used as control groups for cellular viability. DMEM with 10% FBS was added drop-wise using a 1 mL syringe to fill gLL reservoirs. The gLL system was replenished with fresh media every 2–3 days to maintain a healthy cell culture, either drop-wise or via syringe pump. Replacing the media on a timely basis prevents the buildup of cellular waste products within the system. In addition, phenol red in DMEM also helps to indicate any changes in the pH media produced by cellular waste buildup. Representative cell groups were analyzed using a brightfield microscope after 24 h and a ratio of live to dead cells was established to estimate viability upon the different substrate platforms. Viability was calculated via a parameter of survival, *S*, in which optical assessment of cellular attachment and cell-body size upon substrate adhesion is used to denote cell detachment as death [53]. Proliferation was measured using direct cell counting within gLL systems. Glial proliferation at different time points within gLL devices was estimated using the conventional doubling time, defined as:

(2) $$I\left(t\right)=I_{0}2^{\frac{t}{T}}$$

where *I(t)* is the number of cells at time *t* (days), *I*_0_ is the initial number of cells, and *T* is the unitless factor of doubling time. Lastly, MG and ShC morphology were evaluated using the cell shape index (CSI), a dimensionless parameter widely used to quantify the roundness of a cell defined in Equation (3):

(3) $$CSI=\frac{\left(4\pi A_{s}\right)}{P^{2}}$$

where *A_S_* is the surface area (μm^2^) and *P* (μm) is the perimeter of the cell. The value of the CSI ranges from 0 to 1, where values close to 1 represent a perfectly rounded cell and values approaching 0 denote a purely bipolar and elongated cell [9,29,51,54].

### 2.5. Measurement of Cell Migration in the gLL

Cells were seeded into the device at a density of 1.0 × 10^6^ cells/mL in DMEM 10% FBS using a 1 mL syringe and incubated for 24 h. Afterwards, a 50-µL-volume of EGF, VEGF, or BDNF was added to one gLL volume reservoir using a micropipette and an equal volume of serum-free DMEM was added to the opposite gLL reservoir. Molecular transport via diffusion established a linear, quasi-steady-state concentration gradient in the microchannel that stimulates glia chemotaxis. MG and ShC migratory responses were recorded every 30 min by measuring the distance travelled by motile cells, denoted the path length, *PL* [52], for a total of 6 h after quasi-steady-state concentration gradients were established. 

### 2.6. Imaging and Analysis

An inverted epi-fluorescence microscope (Nikon TE2000, Minato, Tokyo, Japan) was used to observe cell behavior over time and to perform optical analysis with a cooled CCD camera (CoolSNAP EZ CCD Camera, Photometrics, Tucson, AZ, USA) using a 20× magnification (Nikon Plan 20×, Morrell Instrument Company Inc., Melville, NY, USA). Microscope images were analyzed for the fluorescent dextran testing using ImageJ [55,56] to determine intensity values (8 bit scale: 1-255) over position and time.

### 2.7. Statistical Analysis

A one-way ANOVA was used to analyze statistical significance among all experimental groups. Each dataset was gathered with a minimum sample size of 15 cells per device, in triplicate. Values are reported using the mean and standard deviation. A post-hoc Tukey test was used to determine pairwise levels of significance between experimental and control groups, where *p*-values < 0.01 compared to control groups are denoted with ** and *p*-values < 0.01 between experimental groups are represented by ꝉꝉ.

## 3. Results

### 3.1. The gLL System

**Figure 4:**
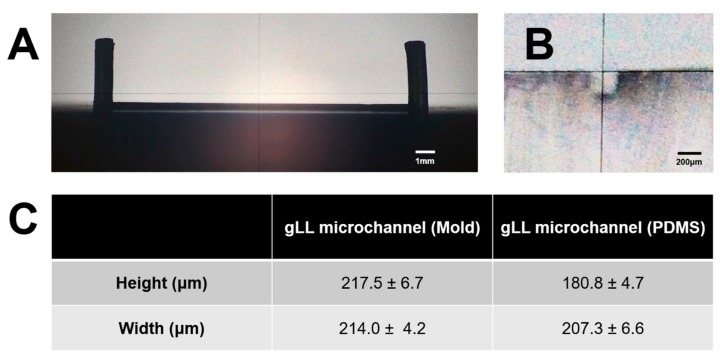
Profile projection of the gLL mold and the PDMS device cast. (**A**) Projection of the gLL mold. The grid is part of the highly precise software used to measure the height and width of the mold. (**B**) Cross-sectional area of the gLL system highlighting the microchannel. (**C**) Average measurements for both the aluminum mold and PDMS microchannel height and width.

The dimensions of the gLL were chosen to represent the anatomical scale of the extracellular environment in which adult glia reside. As per Figure 1, the width of the microchannel approaches the thickness of the retina, through which Müller glia span to establish connections with surrounding cells. Similarly, the microchannel width mimics the thickness of peripheral nerve fibers, composed of bundle of axons, to which Schwann cells aid efficient motor and sensory signal transduction. The fabrication of this device with larger microscale features was performed using a mid-grade, academic CNC instrument with an average cutting feed rate and spindle speed, as described in the literature [57] and summarized in Table 1. As seen in Figure 4, the CNC-fabricated aluminum molds created rectangular microchannels that were also rectangular in cross-section, with an average height of 217.5 ± 6.7 μm and average width of 207.3 ± 6.6 μm, resulting in an average hydraulic diameter of 212.3 μm. Aluminum molds were then used for elastomeric molding using PDMS. As shown, microchannels in PDMS exhibited smaller dimensions of 180.8 ± 4.7 μm and 207.3 ± 6.6 μm, with a D_h_ = 193.1 μm. 

### 3.2. Transport within the gLL Microchannel

The gLL was first filled with PBS to create a fluidically connected system. Fluorescent dextran was then added into one of the reservoirs to diffuse for 24 h within the gLL microchannel. Images were captured at intervals of 100 μm along the microchannel after 24 h, and their average intensity quantified; error bars were calculated using 7% standard error of the mean for at least three data points resulting in a linear distribution of the dextran (R^2^ = 0.9317) along the microchannel length, denoting quasi-steady-state behavior, as shown in Figure 2B. 

### 3.3. Glial Survival

**Figure 5:**
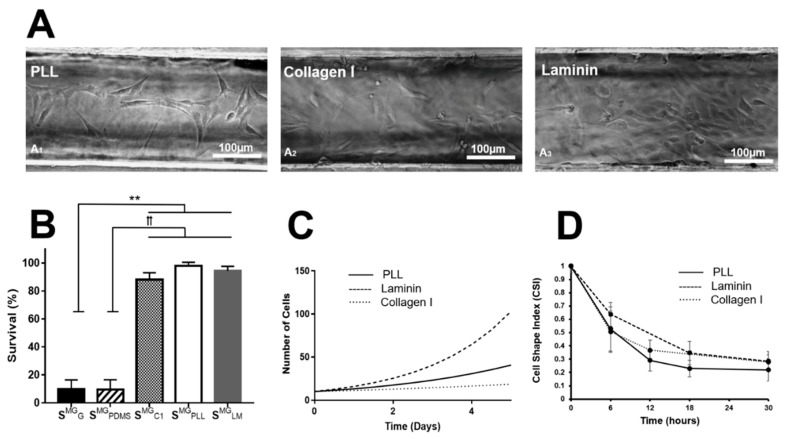
Morphology and proliferation of cultured Müller glia within the gLL system. (**A**) Brightfield images of Müller glia (MG) adhered 24 h after seeding upon within gLL treated with different ECM: (**A1**) Poly-L-Lysine (PLL), (**A2**) Collagen I, (**A3**) Laminin. (**B**) Viability of MG in treated gLL systems 24 h post-seeding. (**C**) MG proliferation within the gLL treated with PLL, Collagen I, and Laminin as a function of time. (**D**) Morphology of adhered MG within treated gLL devices measured by average values of cell shape index (CSI) over time. *p*-values < 0.01 compared to control groups are denoted with ** and *p*-values < 0.01 between experimental groups are represented by ꝉꝉ.

**Figure 6:**
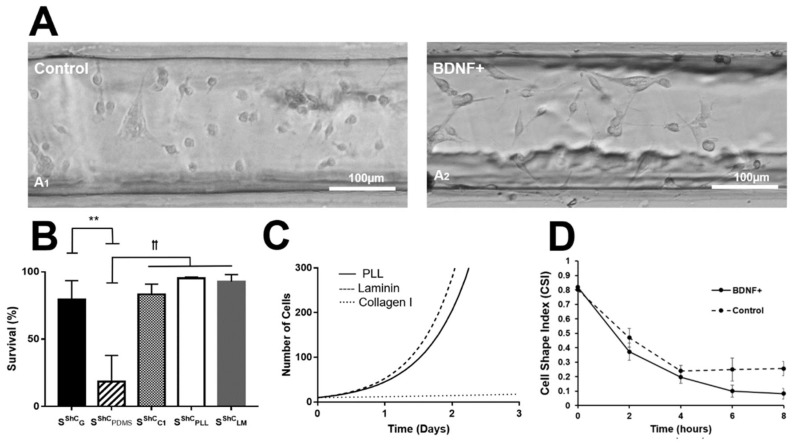
Morphology and proliferation of cultured Schwann Cells within the gLL system. (**A**) Brightfield images of Schwann cells (ShC) adhered 24 h after seeding upon Laminin: (**A1**) ShC in control media, (**A2**) ShC in media containing BDNF (100 ng/mL). (**B**) Viability of ShC within gLL treated with different ECM: Collagen I (C1), Poly-L-Lysine (PLL), and Laminin (LM) 24 h post-seeding. (**C**) ShC proliferation within gLL devices treated with different ECM as a function of time. (**D**) Morphology of adhered ShC within LM-treated gLL with and without BDNF, measured by average cell shape index (CSI) values over time. Statistical significance is represented by * for *p*-values < 0.05, ** for *p*-values < 0.01 with respect to controls and *p*-values < 0.01 between experimental groups are represented by ꝉꝉ.

The micromachined environment of gLL devices was first used to examine the in vitro viability of adult MG and ShC, which have been cultured in the gLL for as long as 96 h. Confluency can challenge glial viability as apoptosis may occur within minimal operational media volume in the microchannel. Cells can be cultured for longer periods provided that media is replenished frequently as the cells proliferate, such as in conditions of serum-starvation [58]. Cells were inserted into untreated gLL devices (i.e., PDMS, glass controls), as well as within gLL devices functionalized with different ECM substrates PLL, LM, or CL1. As seen in Figure 5, MG survival rates upon the control surfaces were relatively low when measured 24 h post-seeding with values of S^MG^_GL_ = 9.67 ± 6.8% upon glass and S^MG^_PDMS_ = 9.47 ± 7.1% upon PDMS. By contrast, MG survival rates within functionalized gLL devices were much higher, measuring S^MG^_PLL_ = 98 ± 2.0% on PLL, S^MG^_LM_ = 94.5% ± 3.2% on LM, and S^MG^_CL1_ = 88.2 ± 5.0% on CL1, 24 h post-seeding. Changes in MG survival were statistically significant between controls and ECM, but not between each ECM-treated device. ShC survival upon PDMS was low with S^ShC^_PDMS_ = 18.4 ± 19.5% but higher upon glass with S^ShC^_GL_ =79.5 ± 13.9%, as per Figure 6. Compared to the control groups, ShC survival was notably higher upon ECM substrates with values of S^ShC^_LM_ = 92.8 ± 5.12% on LM, S^ShC^_PLL_ = 95.2 ± 0.9% on PLL, and S^ShC^_C1_ = 83.28 ± 7.6% on CL1. We note that shadows seen along the channel edges are sometimes cast by the PDMS structure. Measurements were performed using only cells at least one cell diameter away from channel walls where light from the inverted microscope enabled clear distinction of cells. Statistical significance for *p*-values < 0.01 with respect to the glass control group is denoted by ** and *p* < 0.01 with respect to the PDMS group is represented by ꝉꝉ.

### 3.4. Cell Proliferation

Subsequent experiments used the gLL to examine glial proliferation in vitro. Glial growth was tracked upon the three ECM substrates by labeling initial cell populations per square micrometer within gLL devices. MG populations were assessed after 24 h to determine the doubling time upon three matrices. Values of MG doubling time, T, were measured as T^MG^_LM_ = 0.4855, T^MG^_PLL_ = 2.4676, and T^MG^_CL1_ = 5.5875 when seeded upon LM, PLL, and CL1 functionalized surfaces, respectively, as shown in Figure 5. Similarly, ShC seeded the three ECM layers exhibited doubling times of T^ShC^_LM_ = 0.4168 on LM, T^ShC^_PLL_ = 0.4582 on PLL, and T^ShC^_CL1_ = 3.6216 on CL1, as per Figure 6. 

### 3.5. Cell Morphology 

The next set of experiments evaluated changes in adhered glia morphology over time within gLL devices. Cells were allowed to adhere for 2 h prior to testing and monitored over the span of 30 h within the gLL. Figure 5 depicts representative MGs within gLL devices functionalized with different ECM. CSI measurements were used to quantitatively assess changing cell morphology upon the different ECM substrates, where CSI values near 0 represent fully elongated cells and values approaching 1 indicate rounded cells. As seen, all values of CSI decreased over time to exhibit varying average CSI values upon different ECM. Cells upon LM and CL1 were the most elongated 30 h post-seeding with values of CSI^MG^_LM_ = 0.28 ± 0.053 and CSI^MG^_CL1_ = 0.28 ± 0.073, respectively. MG adhered upon PLL illustrated a slightly more elongated morphology with CSI^MG^_PLL_ = 0.22 ± 0.084 at 30 h post-seeding in the gLL. CSI values remained largely constant over the next three days upon all ECM within the gLL. Similarly, Figure 6 depicts images of ShC on LM substrates cultured with BDNF, displaying elongated morphologies after 8 h, with a CSI^ShC^_LM_ = 0.15 ± 0.067 compared to controls. ShC morphology did not significantly change over three days. In addition, changes in CSI from neuroglia within the gLL were compared to the changes in other in vitro conventional, as polystyrene flasks, using the same experimental conditions, as seen in Figure S1. CSI values did not dramatically change at corresponding time points. Media levels in the gLL reservoirs were observed to change by less than 10% every two days in a standard 5% CO_2_ incubator at 37 °C to alleviate concerns of evaporation during experimental setup and testing.

### 3.6. Cell Migration

**Figure 7:**
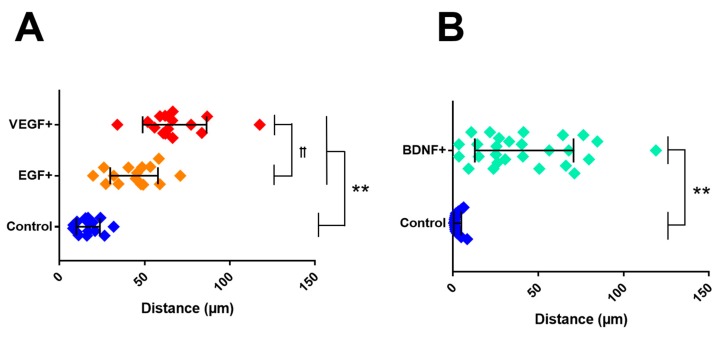
Average distance travelled by glial cells within the gLL system upon growth factor treatment. (**A**) Migration of Müller Glia (MG) within the microchannel upon exposure to EGF, VEGF, and basal conditions (serum-free media). (**B**) Schwann cell (ShC) migration within the microchannel upon exposure to BDNF and basal conditions. Standard deviation is shown within each group and statistical significance is represented by * for *p*-values < 0.05 and ** for *p*-values < 0.01 and *p*-values < 0.01 between experimental groups are represented by ꝉꝉ.

The final experiments of this project examined growth factor-induced migration of MG and ShC glia within gLL devices. MG migration was studied in response to concentration gradients of EGF and VEGF, two key factors for diabetic retinopathy [59,60], while ShC migration was examined in response to gradients of BDNF, critical to nerve regeneration and guidance in peripheral neuropathies [26,27,28,61]. As seen in Figure 7, both glial cell types exhibited chemotactic migration in response to increased concentration gradients. MG migrated in large numbers to growth factor gradients with an average distance, or path length, of PL_VEGF_ = 67.60 µm to VEGF and PL_EGF_ = 43.88 µm to EGF. Both growth factors induced a larger number of MG to migrate compared to control MG groups in DMEM, where cells exhibited an average of PL_CNTL_ = 16.9 µm. ShC illustrated similarly high numbers of motile cells in response to BDNF compared to control, with PL_BDNF_ = 41.82 µm in contrast to PL_CNTL_ = 2.74 µm in control. Further, the distribution of cellular migration data resulted in statistical significance with *p* < 0.01. 

## 4. Discussion

Emerging therapies have begun to explore the native regenerative capabilities of neuroglia to treat a variety of neural disorders in the adult NS [62]. Experimental testing platforms are needed to effectively predict glial response to therapeutic stimuli applied on adult physiological scales to advance translation of glia-based therapies for the treatment of diverse neuropathies. Cell culture of neuroglia in flasks, culture dishes, and well-plates are not successful in accurately recreating microenvironments due to their limited capability in accomplishing laminar flow, build, and retained chemical gradients, unable to offer a constrained environment that resembles the in vivo physiological system. In this work, we report the development of a tunable, microfluidic gLL system that mimics the anatomical scale of the in vivo extracellular environment in which adult glia reside. The benefits of microscale devices with characteristic lengths in the hundreds of microns have been overshadowed by the increasing variety of micron and sub-micron systems developed for high-throughput screening and/or single-cell analyses [63]. Although increasingly miniaturized features offer distinctive advantages [64], the scale and adaptability of the gLL is readily applicable to the adult visual and peripheral NS. The cellular density can be tailored to be studied in the gLL depending on the research objective, as neuroglia density varies across different locations of the nervous system. For instance, Müller glia density is higher in the parafoveal area than in the peripheral section [65]. Likewise, Schwann cell density changes depending upon the length of the axon and its location within the PNS [66]. Furthermore, neurodegenerative diseases such as diabetic retinopathy, age-related macular degeneration, and glaucoma in the visual system and Guillain Barre, Charcot–Marie–Tooth, and multiple sclerosis in the PNS are characterized by large disparities in neuroglia density [67,68,69]. Rendering of neuroglia within the nervous system (Figure 1) is intended to provide a better insight of the cell interaction within characteristic length (*L_c_*). The gLL microscale (Figure 2) is ideal for the study of MG and ShC, in which cell lengths traverse the thickness of adult retina to provide mechanical and synaptic stability [70,71,72] and wrap the axons of multipolar neurons to enable neurotransmission [73,74,75,76], respectively. Importantly, the dimensions of the gLL are well suited to more cost-effective fabrication processes using conventional CNC machining than traditional photolithography [39,52,77]. The well-established processes of CNC milling and machining remain surprisingly overlooked despite the low cost and ultra-durability of the produced milled molds. The milled gLL mold described in this work (Figure 3 and Figure 4) has been used for over 100 casts of PDMS-based devices with minimal wear in the past two years. Moreover, CNC is well-integrated into the undergraduate laboratory training of many technical disciplines [78], presenting an excellent and under-utilized interface to facilitate innovation between clinical researchers and engineers [79]. 

The gLL system is readily integrated with biomedical research as the device can be manually loaded via syringe and placed in an incubator, or incubated *in a* microscope stage, to gather functional data of cell morphology, viability, adhesion, and migration. Furthermore, the gLL system offers a microscale environment that more closely approximates in vivo than well-plates and culture dishes because of its characteristic length (*L_c_*) in hundreds of microns and the smaller scale of microliter volume it uses to surround cells. The gLL additionally enables quantification of chemotaxis in long-lasting concentration gradients, as well as the study of cellular morphology and proliferation in an environment similar in scale to in vivo conditions. MG and ShC were observed to adhere, proliferate, and thrive within the confined environment of the gLL for days. Survival in all ECMs for both MG and ShC was above 90% (Figure 5 and Figure 6), demonstrating the ability of larger microscale systems to provide critical data denoting differences in adult glia viability within confined microenvironments (reviewed in [80]). This work additionally measured reproducible changes in cell adhesion morphology upon different ECM, where neuroglia showed extended processes ranging above 100 µm in length. (Figure 5 and Figure 6). The data illustrate gLL capabilities to effectively examine adhesion, important when analyzing the compatibility of newly synthesized implant coatings and 3D grafts currently being developed for promising cell replacement therapies [20]. Adhesion patterns were optically assessed to denote changes in cellular morphology via CSI, which was used to describe the extension of processes and larger adhesion areas upon ECM substrates. Lastly, experiments also illustrated that confined microenvironments had minimal impact on MG and ShC proliferation. This is clinically significant because glia generate both protective [81,82] and toxic [82,83] effects in response to acute injury, often hyper-proliferating and forming glial scarring that retards healing. Thus, gLL-based testing can aid the study of MG hyper-proliferation central to reactive gliosis and progression of retinopathies that affect an increasing number of adults worldwide [84,85,86,87,88], as well as ShC contributions to changes in neurotransmission post-spinal cord injury [25,89]. 

The gLL can be used to examine chemotactic glial migration, which is paramount to many regenerative therapies in which transplanted MG and ShC must navigate extracellular spaces to repair neurons and isolate damaged cells [90,91,92,93,94]. Data illustrated that both MG and ShC migrated longer distances in response to VEGF and EGF, or BDNF, respectively, with respect to basal conditions. The VEGF growth factor is strongly implicated in angiogenesis, where it is largely secreted by endothelial cells [95]. Upregulation of VEGF in the retina leads to excessive vascular permeability and infiltration of harmful molecules, which stimulate proliferation and migration of MG [96]. Similarly, EGF has been reported to enhance migratory behavior of Müller glia, which re-enter the cell cycle and migrate towards the subretinal space to disrupt retinal laminae [97]. BDNF is expressed after peripheral nerve injury to stimulate axonal regeneration. While these reparative mechanisms remain only partially understood, ShC are well-known to migrate towards the injured site to aid neural repair [98]. MG exhibited path lengths that were up to three times larger than those in basal conditions in response to growth factor signaling, while ShC exhibited path lengths 20 times larger than those measured using control conditions (Figure 7). Data describing glia chemotactic behavior are meaningful for the development of pharmacological treatments that induce or retard MG migration in diabetic retinopathy and ShC migration in peripheral neuropathies, as well as glial scarring across the NS [99]. 

In conclusion, the highly tunable microfluidic gLL system provides a versatile platform for translational study of glia in regenerative therapies. The ability to quantify biological parameters within the gLL, offers an effective and reliable alternative to photolithography to study neuroglia and their local microenvironment, which ranges on the tens to hundreds of microns, using a low-cost and easily fabricated system. Data confirmed the ability to image and analyze the adhesive, proliferative, and migratory behaviors of MG and ShC using controlled stimuli within microenvironments similar in scale to adult glia physiology. For instance, Müller glia extend their processes to create anatomical barriers at the inner and outer limiting membrane retina [100]. Likewise, Schwann cells display numerous processes, used to wrap around neuronal axons and cap axon terminals [66,101]. These morphological characteristics resemble adhesion patterns of glia within the in vivo environment. In addition, migration assays displayed low levels of basal migration, but increased movement in the presence of growth factors (e.g., VEGF, EGF, and BDNF), similar to glial response in neurodegenerative disorders, reported by many research groups [102,103,104].

Importantly, the gLL was manufactured using traditional CNC machining, eliminating the dependence on specialized photolithography techniques and reducing the cost and barriers to device design and operation. 

## Figures and Tables

**Table 1:** CNC machining parameters for the aluminum gLL mold. The gLL mold was created using a 3-axis TRAK DPM SX2P and ProtoTRAK SMX computerized numeric control (CNC) milling machine. A partial list of axis value parameters is provided per the manufacturer specifications.

Axis Travel	Parameter
X-, Y-, and Z-axes travel (cm)	81.3 × 40.6 × 59.7
Table working area (cm)	125 × 23
Table weight (kg)	~1452
Feed per tooth (fz)	0.0965
Spindle motor (kw)	2.2371
Spindle speed (rpm)	40–600, 300–5000
Cutting feed rate (mm/min)	1346
Position precision (mm)	0.127
Position repeatability (mm)	0.127
Lubrication	Yes

## Supplementary Materials

The following are available online at https://www.mdpi.com/2072-666X/10/8/513/s1, Figure S1. Morphology of cultured Müller glia and Schwann Cells within the gLL system and conventional culture flasks. (A) Morphology of adhered MG within treated gLL devices measured by average values of Cell Shape Index (CSI) over time. (B) Morphology of adhered MG within treated polystyrene flasks. (C) Morphology of adhered ShC within LM-treated gLL with and without BDNF. (D) Morphology of adhered ShC within LM-treated polystyrene flasks.
