# Supplementary material for: A Milled Microdevice to Advance Glia-Mediated Therapies in the Adult Nervous System
Source: Micromachines (Basel). 2019 Jul 31;10(8):513. doi: 10.3390/mi10080513 (PMC6723365; doi:10.3390/mi10080513)
Supplement: Supplementary file 1 [file micromachines-10-00513-s001.pdf]

Supplementary Materials: A Milled Microdevice to Advance Glia-Mediated Therapies in the Adult Nervous System

Juan S. Peña, Denise Robles, Stephanie Zhang and Maribel Vazquez

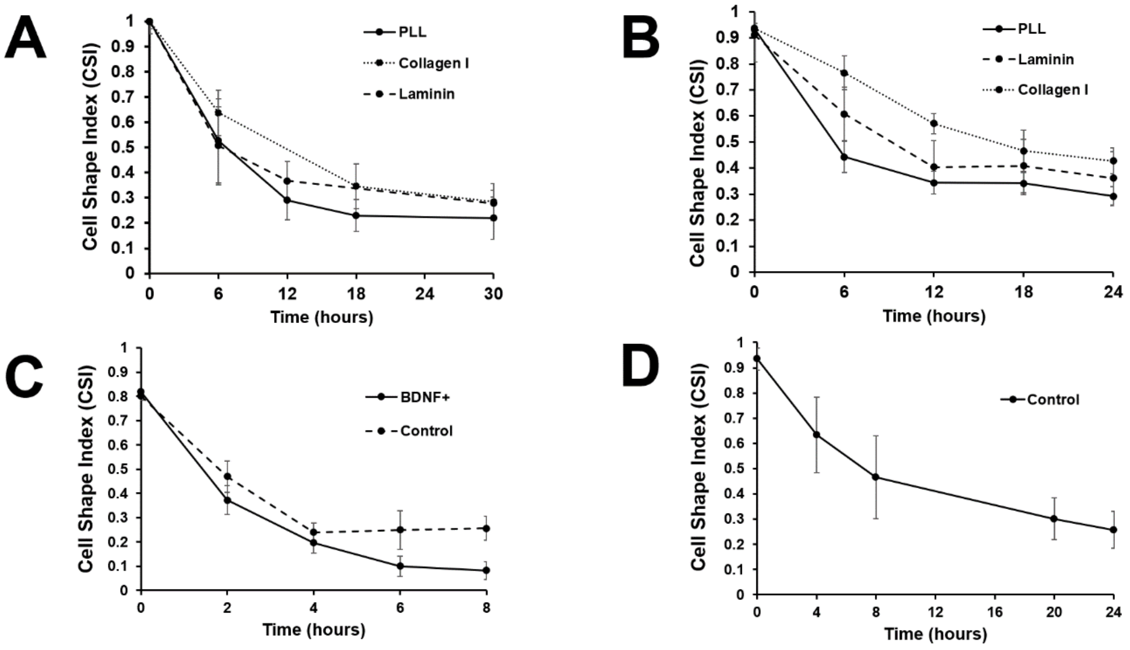

Figure S1. Morphology of cultured Müller glia and Schwann Cells within the gLL system and conventional culture flasks**.** **(A)** Morphology of adhered MG within treated gLL devices measured by average values of Cell Shape Index (CSI) over time **(B)** Morphology of adhered MG within treated polystyrene flasks **(C)** Morphology of adhered ShC within LM-treated gLL with and without BDNF. **(D)**, Morphology of adhered ShC within LM-treated polystyrene flasks.
